# Antioxidative, Antibacterial, and Food Functional Properties of the Half-Fin Anchovy Hydrolysates-Glucose Conjugates Formed via Maillard Reaction

**DOI:** 10.3390/molecules21060795

**Published:** 2016-06-20

**Authors:** Ru Song, Peiyu Yang, Rongbian Wei, Guanqiang Ruan

**Affiliations:** 1 Zhejiang Provincial Key Laboratory of Health Risk Factors for Seafood, College of Food Science and Pharmacy, Zhejiang Ocean University, Zhoushan 316022, China; peiyuyang454@163.com (P.Y.); guanqiangruan@163.com (G.R.); 2 College of Marine Science and Technology, Zhejiang Ocean University, Zhoushan 316022, China; rbwei@zjou.edu.cn

**Keywords:** half-fin anchovy hydrolysates, Maillard reaction, antioxidative activity, antibacterial activity, functional property

## Abstract

The antioxidative, antibacterial, and food functional properties of the half-fin anchovy hydrolysates (HAHp)-glucose conjugates formed by Maillard reaction (MR) were investigated, respectively. Results of sugar and amino acid contents loss rates, browning index, and molecular weight distribution indicated that the initial pH of HAHp played an important role in the process of MR between HAHp and glucose. HAHp-glucose Maillard reaction products (HAHp-G MRPs) demonstrated enhanced antioxidative activities of reducing power and scavenging DPPH radicals compared to control groups. HAHp-G MRPs produced from the condition of pH 9.6 displayed the strongest reducing power. The excellent scavenging activity on DPPH radicals was found for HAHp(5.6)-G MRPs which was produced at pH 5.6. Additionally, HAHp(5.6)-G MRPs showed variable antibacterial activities against *Escherichia coli*, *Pseudomonas fluorescens*, *Proteus vulgaris*, *Pseudomonas aeruginosa*, *Staphylococcus aureus*, *Bacillus subtilis*, *Bacillus megaterium*, and *Sarcina lutea*, with the MIC values ranging from 8.3 to 16.7 μg/mL. Result of scanning electron microscopy (SEM) on *E. coli* suggested that HAHp(5.6)-G MRPs exhibited antibacterial activity by destroying the cell integrity through membrane permeabilization. Moreover, HAHp(5.6)-G MRPs had excellent foaming ability and stability at alkaline conditions of pH 8.0, and showed emulsion properties at acidic pH 4.0. These results suggested that specific HAHp-G MRPs should be promising functional ingredients used in foods.

## 1. Introduction

Maillard reaction (MR), also called non-enzymatic browning, refers to the interaction initiated between an amino group of amino acids, proteins, or any nitrogenous compound, and the carbonyl moiety of reducing sugars, aldehydes, or ketones [1]. MR is the main reaction responsible for taste, aroma, color, texture, and biological activity of foods during food processing and preservation [2,3,4,5]. Since it was first described at the beginning of the 20th century [4], MR is still extensively studied, due to multiple biological activities of the Maillard reaction products (MRPs).

The antioxidative activity of MRPs was reported in 1954 [6]. Up to date, numerous studies have found the strong antioxidative activities of MRPs, which originated mainly in three categories: (1) model mixtures by heating solutions of single amino acid and reducing sugar, such as lysine/glycine–glucose [7], lysine–fructose/ribose [8]; (2) glycation of protein or hydrolysates, e.g. casein [9,10], bovine lactoferrin [11], porcine plasma protein [12], whey protein [13], and soybean protein hydrolysates [14]; and (3) extracts from foods containing high MRPs levels, e.g. coffee or roasting products [8,15,16,17]. In addition, MRPs produced from the reaction of lysozyme or chitosan in model systems, like lysozyme-galactomannan conjugates [18], chitosan-lysozyme conjugates [19], and ε-polylysine–chitosan MRPs [20], indicated higher antimicrobial activities against food pathogenic bacteria or spoilage bacteria than chitosan or lysozyme alone.

The excellent techno-functional capacity of proteins may be of considerable interest to the food industry, which would determine their potential applications in foods. It has been reported that the functional properties of proteins or saccharides, such as the solubility, heat stability, emulsifying and foaming properties, were found enhanced via MR for glycated β-lactoglobulin [4,21,22,23], milk protein [24], egg white [25], peanut protein [26], and chitosan [27]. Although a reduction of the nutritive value, especially lysine loss, and possible formation of toxic compounds harmful to human health produced in MR [28,29,30], the food proteins modified by MR could give rise to fewer safety issues compared to chemically-modified counterparts [31]. In recent several studies, Oh and colleagues described the dietary MRPs and their fermented products reduced cardiovascular risk in an animal model [32]. Li et al. firstly reported that the acute LD_50_ of glucose–cysteine MRPs in rats was 6.81 g/kg, and repeated 90-day administration of less than, or equal to, one-fifth LD_50_ produced no significant toxicities in experimental animals [33]. Consequently, MR is considered as a promising and effective way to produce bioactive and functional ingredients.

Half-fin anchovy (*Setipinna taty*), one kind of small marine fishes, can be captured along the coastal waters in China. In our previous studies, we reported the peptic hydrolysates of half-fin anchovy (HAHp) and their thermal products demonstrated antioxidative, antibacterial, and antiproliferative activities [34,35]. However, to our knowledge, there are few studies about HAHp glycated with sugars through MR and the properties of the resulting products. The goal of this study is to investigate the influence of different pH values for the production of HAHp/glucose MRPs (HAHp-G MRPs) and their varied sugar and amino acid contents, browning indices, molecular weight distributions, and antioxidative activities. Subsequently, the antibacterial activities of the bioactive HAHp-G MRPs were evaluated, as well as their characteristics of foaming and emulsifying properties in different pH ranges. This knowledge could provide information for the potential application of HAHp-G MRPs used in foods.

## 2. Results and Discussion

### 2.1. Characteristics of Sugar and Amino Acid Contents and Browning Index of HAHp-G MRPs

**Figure 1:**
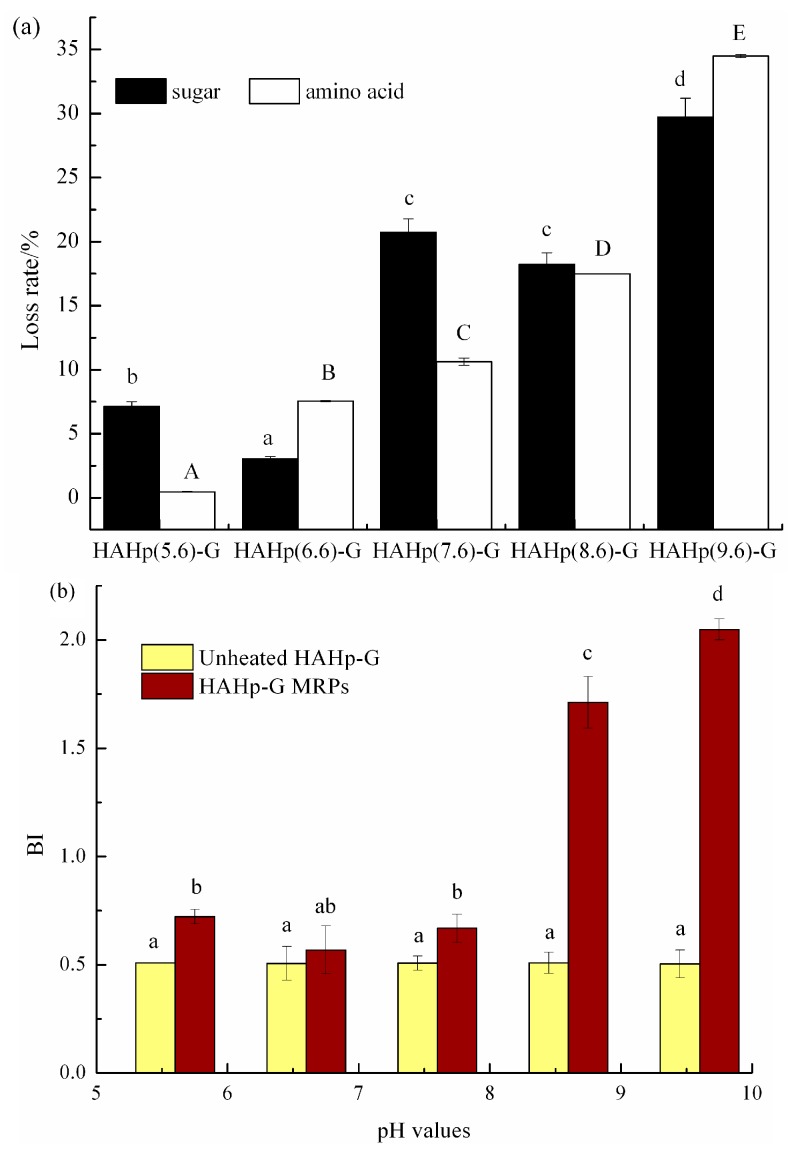
Comparison of (**a**) the loss rates of sugar and amino acid contents, and (**b**) the BI between unheated HAHp-G and HAHp-G MRPs produced at different pH values. Data were presented as mean ± SD (n = 3). Different letters (a–d) and capital letters (A–E) in Figure 1a represented significant difference in sugar loss rate and amino acid loss rate, respectively (*p* < 0.05). Different letters (a–d) in Figure 1b represented significant difference in BI (*p* < 0.05).

During the MR, amino acids could interact with reducing sugars and, therefore, result in the decreased amounts of amino acids and reducing sugars [1]. The formation of advanced MRPs, which are characteristic of visible absorbance at 420 nm, will be responsible for the development of brown color [36]. The higher browning index (BI) suggests the greater amount of advanced MRPs [37]. Figure 1a shows the loss rates of sugar and amino acids contents were increased with the elevated initial pH values. On the contrary, the BI of HAHp-G MRPs was enhanced with the increased initial pH values. Sharply increased BI was observed at the initial pH of HAHp over 8.6 (Figure 1b). In the MR, higher pH would favor the formation of reductone via furfural production from the Amadori products and, therefore, lead to dark color development [38]. In addition, the caramelization reactions could occur simultaneously with the MR, and contribute to overall non-enzymatic browning, especially in the alkaline pH ranges [39]. Results of Figure 1 confirmed that the formation of MRPs derived from HAHp and glucose were with the initial pH values ranging from 5.6 to 9.6, especially at alkaline pH conditions.

### 2.2. Molecular Weight Distribution of HAHp-G MRPs

**Figure 2:**
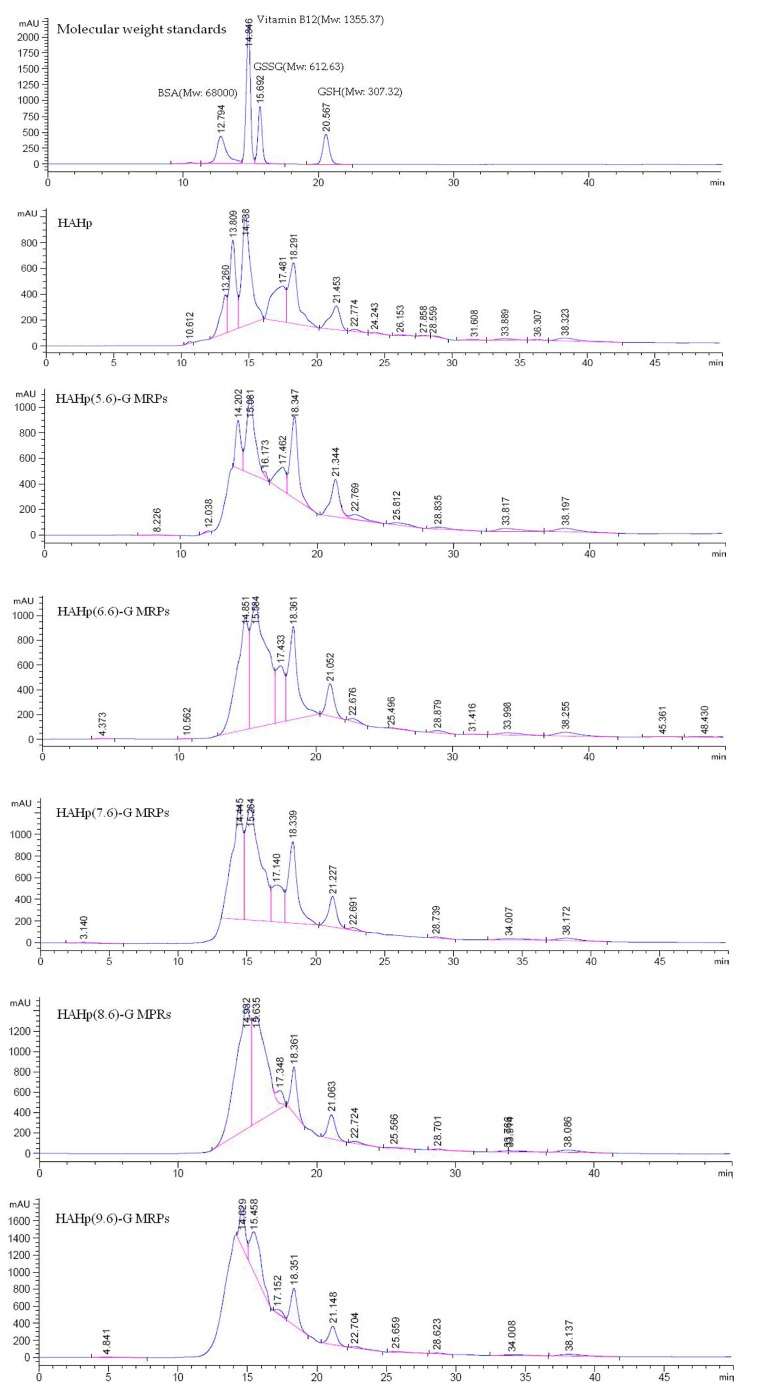
Elution profiles of HAHp and HAHp-G MRPs (prepared at pH 5.6, 6.6, 7.6, 8.6, and 9.6) on HPLC system using a PL aquagel-OH 30 column (7.5 × 300 mm, 8 μm), eluted with 50 mM sodium phosphate buffer (pH 5.8) under a flow rate of 0.5 mL/min.

It is reported that peptide degradation and cross-linking simultaneously occurred in the MR [40]. In this sense, smaller peptides or free amino acids might tend to form because of peptides/proteins degradation. On the other hand, larger molecules could be yielded due to peptides/amino acids cross-linking [41]. As shown in Figure 2, HAHp mainly composed of small peptides with molecular weight below 1500. A few contents of large peptides or proteins were also observed in HAHp. However, different products of HAHp-G MRPs displayed various patterns in molecular weight distributions. A slight change of molecular weight distribution was observed for HAHp(5.6)-G MRPs, which was prepared at pH 5.6. By contrast, the dramatic change of molecular weight distribution was observed for HAHp(9.6)-G MRPs (prepared at pH 9.6). This was consistent with the loss rates for sugar and amino acid, as well as the BI for HAHp-G MRPs in alkaline pH conditions. Furthermore, Figure 2 shows that larger molecules in HAHp, such as the fraction with retention time of 13.260 min, were disappeared in HAHp-G MRPs produced at pH above 6.6. Similarly, the amount of smaller molecules in HAHp(9.6)-G MRPs, such as the fraction with the retention time of 21.148 min, was dramatically decreased. The results revealed that degradation of large proteins or peptides and hence generation and cross-linking of small peptides or free amino acid happened simultaneously during the MR of HAHp and glucose.

### 2.3. Antioxidative Activity of HAHp-G MRPs

**Figure 3:**
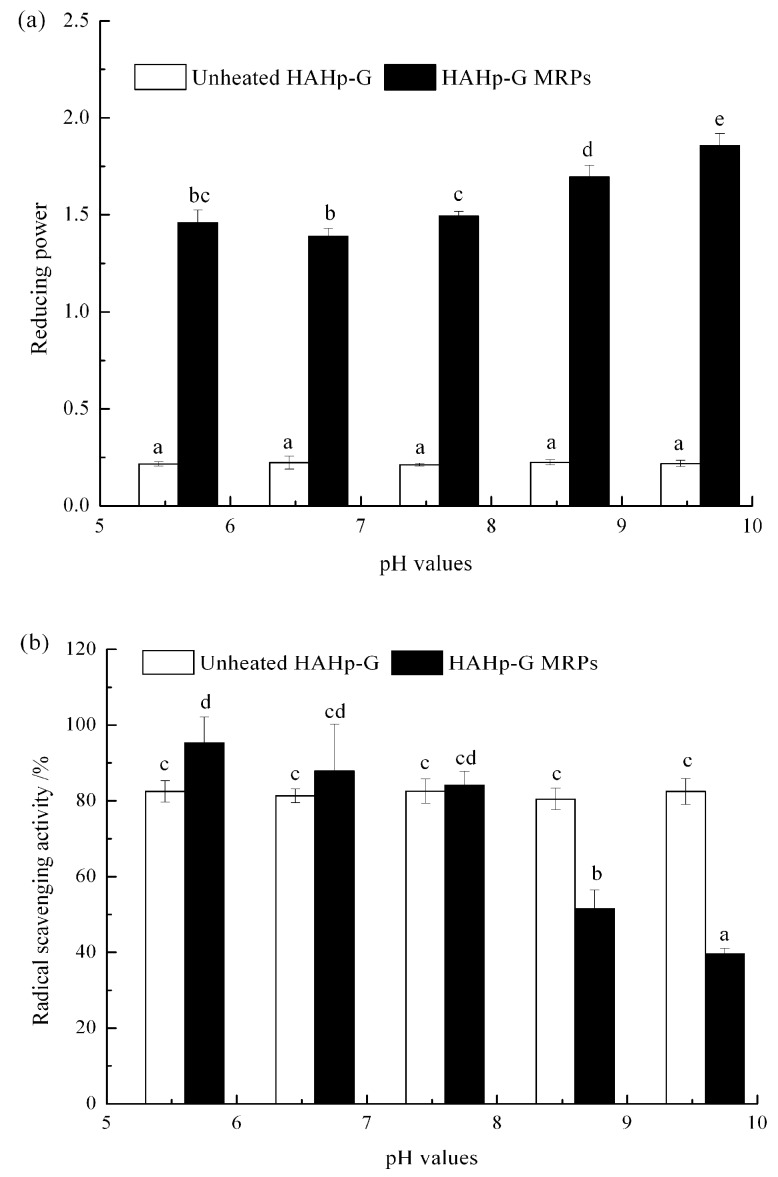
Antioxidative activity of HAHp-G MRPs produced under various pH values. (**a**) Reducing power, and (**b**) radical scavenging activity on DPPH radical. Results were presented as mean ± SD (n = 3). Different letters (a–e) in Figure 3a represented significant difference in reducing power (*p* < 0.05). Different letters (a–d) in Figure 3b represented significant difference in radical scavenging activity on DPPH radical (*p* < 0.05).

The initial pH strongly influences the proportion of amino acids in the unprotonated form and, thus, determines the augment of MR [42]. HAHp-G MRPs prepared at different pH values showed different antioxidative activities in terms of reducing power and DPPH radicals scavenging (Figure 3). The reducing power of HAHp-G MRPs was dramatically enhanced along with increased pH values (Figure 3a). HAHp(9.6)-G MRPs, which were produced at initial pH of 9.6, displayed near eight-fold reducing power capacities than its control group (unheated HAHp(9.6)-G). Noticeably, when it comes to the DPPH radicals scavenging rate, the HAHp(5.6)-G MRPs, prepared at pH 5.6, reached to 95.30% (Figure 3b), which was much higher than those of the control (unheated HAHp(5.6)-G) (82.48%), the HAHp(8.6)-G MRPs (51.54%), and the HAHp(9.6)-G MRPs (39.57%).

Hydroxyl and pyrrole groups of advanced MRPs are considered as effective electron donors to increase reducing power, or transfer DPPH radicals to a stable DPPH–H molecule [43,44,45]. Results from Figure 3 suggested that alkaline condition could contribute to the enhanced reducing power of HAHp-G MRPs, while the initial pHs ranging from 5.6 to 7.6 could be an important factor for generation of DPPH radical scavenger in HAHp-G MRPs. It has been reported that the improved antioxidant activities of MRPs could be related with their high molecular weight and low molecular weight components [41]. In this study, HAHp(9.6)-G MRPs demonstrated the highest reducing power ability and the most dramatically changed molecular weight distribution than the other HAHp-G MRPs (shown in Figure 2 and Figure 3). We supposed that the increased reducing power of HAHp(9.6)-G MRPs could be attributed to their relatively high molecular weight components, whereas the relatively low molecular weight components might be accountable for the enhanced DPPH radicals scavenging activity of HAHp(5.6)-G MRPs.

In the process of MR, racemization of L-amino acid residues to d-amino acid takes place at alkaline pH and high temperature [46]. D-amino acids are considered as less hydrolyzed by proteases [47]. In addition, the carbanion formed under alkaline pH can also undergo β-elimination reaction to produce dehydroalanine, leading to the less digestibility of protein and bioavailability of lysine [47]. In addition, many diseases, such as diabetes and Alzheimer's disease, are related with endogenous advanced glycation end products (AGEs) [48,49]. Endogenous AGEs are also formed by MR during the heat processing of foods [33]. Food source AGEs intake might be accumulated in human body as endogenous AGEs. Therefore, the desired MRPs should have high bioactive activity, but fewer negative effects (e.g., loss of essential amino acids or the formation of potent hazardous compound [28,29,30]. Inferring comprehensively from the results of Figure 1, Figure 2 and Figure 3, we suggested that HAHp (5.6)-G MRPs should be a type of desirable MRPs, which demonstrated not only strong DPPH radical scavenging activity and reducing power capacity, but also much lower BI than HAHp(8.6)-G and HAHp(9.6)-G. It also indicated that minimization of MR between HAHp(5.6) and glucose could, thereafter, relieve the possible negative effects of MRPs.

### 2.4. Antibacterial Activity of HAHp(5.6)-G MRPs

#### 2.4.1. Antibacterial Spectra and the Minimal Inhibitory Concentration (MIC)

As shown in Table 1, HAHp(5.6)-G MRPs demonstrated a broad antibacterial spectrum. *E. coli* was most sensitive to HAHp(5.6)-G MRPs among all the tested bacteria, with inhibitory zone of 28.2 mm diameter. The MIC values of all tested strains ranged from 8.3 to 16.7 μg/mL. The results indicated that the antibacterial effects of HAHp(5.6)-G MRPs were stronger than those of the non-MR treated HAHp against *E. coli, P. fluorescens, P. vulgaris, P. aeruginosa, S. aureus, B. megaterium,* and *S. lutea*, whose MICs fell in the range of 28.38–113.59 μg/mL. This was reported in our previous study [35]. Hence, it can be inferred that MR should be an effective approach for HAHp to improve its antibacterial activity. In a lot of literature it can be found the reports of increased antibacterial and antioxidant activities for the MRPs of proteins, peptides or hydrolysates, and these MRPs were utilized for a variety of foods storage. For example, lysozyme-chitosan conjugates demonstrated greatly enhanced bactericidal action against *Escherichia coil* K-12 [19], and chitosan/glucose MRPs with excellent antioxidant activity and similar antibacterial activity compared to chitosan and can increase the shelf life of lamb meat, pork meat, ground chicken breast, table grapes, and shiitake mushroom [50,51,52,53]. Our results implied that HAHp(5.6)-G MRPs could be a promising novel preservative used for food storage.

#### 2.4.2. Scanning Electron Microscopy (SEM)

**Figure 4:**
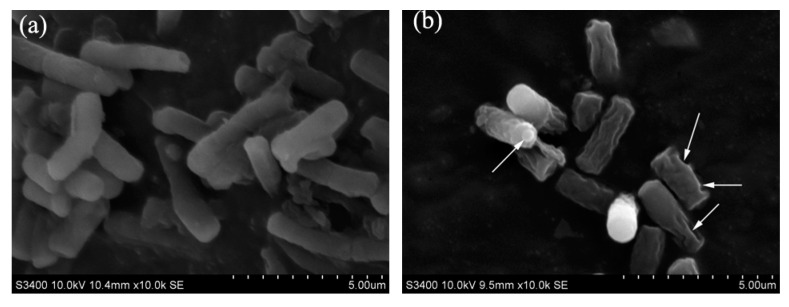
SEM of *E. coli* treated with HAHp(5.6)-G MRPs at 37 °C for 3 h. (**a**) Control group, and (**b**) treated group.

The changes of cell membrane of *E. coli* treated with HAHp(5.6)-G MRPs were shown in Figure 4. The control group displayed typical surface of *E. coli* cells, without apparent cellular debris (Figure 4a). In contrast, lots of morphological changes for *E. coli* were observed after treated with HAHp(5.6)-G MRPs (Figure 4b). The cell surface of *E. coli* was greatly rougher than that of the control. Furthermore, many pores were found in the bacterial surface. Results of Figure 4b suggested that HAHp(5.6)-G MRPs could destroy the cell integrity, likely by causing membrane permeabilization. Similar to our results, Liang et al., reported the ultimately disrupted bacterial cell membranes of *E. coli* with the release of cellular cytoplasm, after treated by ε-polylysine/chitosan MRPs [20]. It is reported that the MRPs endow excellent surfactant properties and, therefore, might destabilize the outer membrane and inhibit the growth of bacterial cells [18]. Additionally, Rufian-Henares and De la Cueva described the Mg^2+^-chelating property could be responsible for the antibacterial activities of coffee melanoidins (one kind of advanced MRPs) [16]. Hauser, Müller, Sauer, Augner and Pischetsrieder found the antimicrobial activity of D-ribose and L-lysine MRPs was, at least partially, mediated by H_2_O_2_ [54]. However, the antibacterial mechanism of MRPs has not been elaborated completely. In subsequent study, we will further investigate the mechanism of HAHp (5.6)-G MRPs against bacteria and identify the active compounds.

### 2.5. Functional Properties

**Figure 5:**
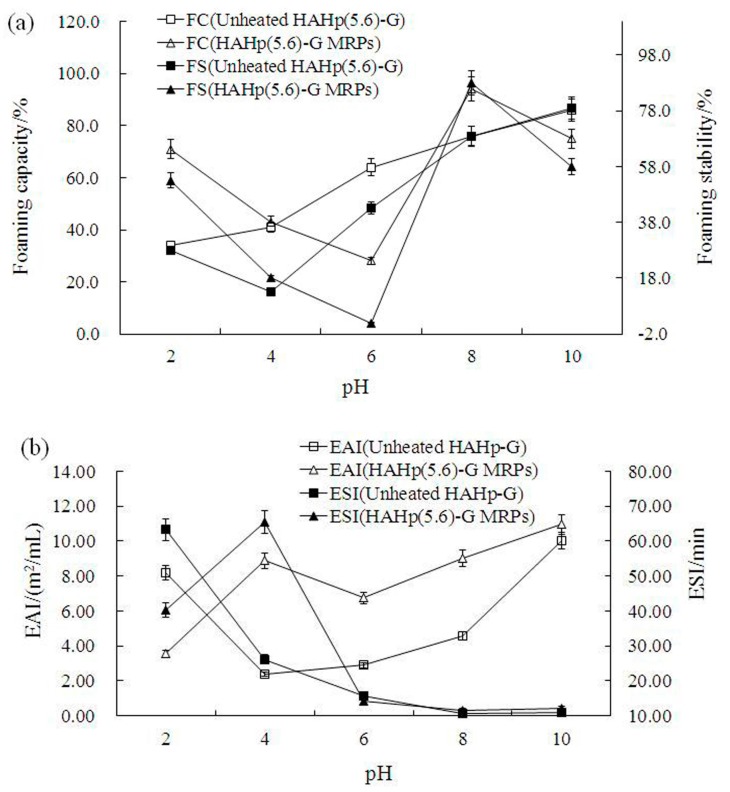
Functional properties of HAHp(5.6)-G MRPs at different pH values. (**a**) Foaming capacity (FC) and stability (FS), and (**b**) emulsifying capacity index (EAI) and emulsion stability index (ESI). Results are presented as mean ± SD (n = 3).

The colloidal behavior of proteins is influenced by pH and consequently affects the techno-functionality of proteins and their incorporation into various foods [11]. In this study, the functional properties including foaming capacity (FC), foaming stability (FS), emulsifying activity index (EAI), and emulsion stability index (ESI) for HAHp(5.6)-G MRPs and unheated HAHp(5.6)-G, both having undergone the same pH condition in a pH range were investigated. As shown in Figure 5a, the FC of HAHp(5.6)-G was enhanced with the increased pH values. The lowest FS for HAHp(5.6)-G was observed at the pH 4.0 (12.5%), whereas the lowest and highest FC of HAHp(5.6)-G MRPs were found at pH 6.0 (27.5%) and pH 8.0 (94.5%), respectively. Similar to the result of FC, HAHp(5.6)-G MRPs demonstrated the lowest and the highest FS at pH 6.0 (2.0%) and pH 8.0 (87.5%), respectively. The nature of the film in the foam and the extent of protein-protein or peptides interaction within the matrix could determine the FS of proteins or peptides [55].

Results of Figure 5a indicated that HAHp(5.6)-G MRPs had relatively stronger FC and FS at the pH value of 8.0 than at other pH conditions. This was likely ascribed to the repulsion of MRPs via ionic repulsion and the steric protection of inner phase material against aggregation and coalescence [55]. Similar to our study, the increased stability of protein foams was reported for β-lactoglobulin/glucose or milk protein/saccharide conjugates through MR [4,21,24]. The formation of thick, viscoelastic MRPs layers at the air/liquid interfaces could promote foam stability [56,57].

As shown in Figure 5b, except for at the pH 2.0, HAHp(5.6)-G MRPs exhibited stronger EAI than unheated HAHp(5.6)-G in the pH range from 4.0 to 10.0, The highest ESI of HAHp(5.6)-G MRPs was recorded at pH 4.0 (65.37 min), which was 2.5-fold of that by unheated HAHp(5.6)-G (26.11 min). However, both HAHp(5.6)-G MRPs and unheated HAHp(5.6)-G showed small ESI values when the pH was above 4.0. Results shown in Figure 5b indicated the strong emulsion properties, including EAI and ESI, for HAHp(5.6)-G MRPs and unheated HAHp(5.6)-G were at pH 4.0 and pH 2.0, respectively. Similar to our results, Song *et al*. reported the lysozyme–HMC (high molecular weight-type chitosan) MRPs had greatly improved emulsion stability at acidic pH [19]. ESI reflects the ability of proteins or peptides to impart strength to emulsion for resistance against coalescence upon storage [58]. In this sense, the emulsion stabilized by HAHp(5.6)-G MRPs was also remarkably resistant to coalescence under acidic conditions. It should be noted that HAHp(5.6)-G MRPs demonstrated excellent emulsion property at relatively mild acidic conditions compared to unheated HAHp(5.6)-G. Consequently, the emulsion properties of HAHp(5.6)-G MRPs may be of considerable interest to food industry.

## 3. Materials and Methods

### 3.1. Materials

Half-fin anchovy (*Setipinna taty*) were purchased from a local fish market in Zhoushan, China. The fish were stored on ice during transportation to the laboratory and separately stored in a polyethylene bag at −20 °C until use. The reagent, 1,1-diphenyl-2-picrylhydrazayl (DPPH), was obtained from Sigma Chemicals Co. (St. Louis, MO, USA). Agar medium was purchased from Tianhe reagent company (Hangzhou, China). Indicator bacterial strains, *Escherichia coli* (CGMCC 1.1100), *Pseudomonas fluorescens* (CICC 20225), *Proteus vulgaris* (CICC 20049), *Pseudomonas aeruginosa* (CMCC 10104), *Staphylococcus aureus* (CMCC 26003), *Bacillus subtilis* (CMCC 63501), *Bacillus megaterium* (CICC 10324), and *Sarcina lutea* (CGMCC 1.258) were purchased and stored at the College of Food Science and Pharmacy, Zhejiang Ocean University. All other reagents used in the experiments were of analytical grade.

### 3.2. Preparation of HAHp-G MRPs

HAHp were prepared by the method described in our previous study [35]. The pH values of HAHp were adjusted to 5.6, 6.6, 7.6, 8.6, and 9.6, respectively, were meanwhile mixed with glucose (0.25%, w/v), respectively. The mixtures were then heated separately at 100 °C for 60 min to prepare HAHp-G MRPs. All of these resulting HAHp-G MRPs were thereafter centrifuged at 10,000*× g* for 20 min (Himae CF 16 RX versatile compact centrifuge, Tokyo, Japan). The supernatants were collected and stored at −20 °C until use.

### 3.3. Sugar and Amino Acid Content of HAHp-G MRPs

The sugar content of unheated HAHp-G mixtures and HAHp-G MRPs were measured using an HPLC method with some modifications [59], with glucose as the standard. The amino acid contents of unheated HAHp-G mixtures and HAHp-G MRPs were determined according to the ninhydrin reaction method described in our previous report [34], with l-serine as a standard. The content of sugar or amino acid was expressed as mg per mL of sample. The loss rate of sugar or amino acid content was calculated using the following Equation (1):

loss rate (%) = [(A_B_ − A_S_/A_B_] × 100 (1)

where A_B_ and A_S_ represented the content of sugar or amino acid in unheated HAHp-G mixtures and HAHp-G MRPs, respectively.

### 3.4. Measurement of BI

The development of brown color is usually used to assess the extent of MRPs [60]. The browning intensity (BI) of HAHp-G MRPs was measured according to Samaras, Camburn, Chandra, and Gordon [61]. Briefly, the BI of HAHp-G MRPs was assayed at a wavelength of 420 nm in a spectrophotometer with cuvettes of 1 cm optical length. The BI of unheated mixtures of HAHp and glucose (unheated HAHp-G) was also determined for comparison.

### 3.5. Molecular Weight Distribution of HAHp-G MRPs

Different HAHp-G MRPs were fractioned on a PL aquagel-OH 30 column (7.5 × 300 mm, 8 μm) equilibrated with 50 mM sodium phosphate buffer (pH 5.8) individually on an HPLC system (Agilent 1260 Infinity, Waldbronn, Germany). The elution rate was 0.5 mL/min. The different fractions were measured by absorbance at 220 nm. Molecular weight (Mw) standards: bovine serum albumin (BSA) (Mw: 68000), vitamin B_12_ (Mw: 1355.37), oxidized glutathione (GSSG) (Mw: 612.63) and reduced glutathione (GSH) (Mw: 307.32) were used to calibrate the molecular weight distribution.

### 3.6. Determination of Antioxidative Activity of HAHp-G MRPs

In this study, we used two indirect electron transfer methods, namely reducing power and DPPH radical-scavenging assay to evaluate the antioxidative activity of HAHp-G MRPs produced at pH 5.6, 6.6, 7.6, 8.6, and 9.6 respectively. Meanwhile, the antioxidative activity of unheated HAHp-G was assayed for comparison.

#### 3.6.1. Reducing Power Assay

The reducing power of HAHp-G MRPs was measured according to the method described by Oyaizu [62] with slight modifications. Briefly, 100 μL of HAHp-G MRPs were diluted into 1.0 mL with distilled water, and mixed with 1.0 mL of 0.2 M sodium phosphate buffer (pH 6.6), followed by the addition of 1 mL of 1% potassium ferricyanide. The reaction mixtures were incubated at 50 °C for 20 min, and then cooled to room temperature. After adding 1 mL of 10% trichloroacetic acid, the mixture solutions were centrifuged at 3000*× g* for 10 min. Two microliters of supernatant was removed and blended with 2 mL of distilled water and 0.8 mL of 0.1% ferric chloride. After kept at room temperature for 10 min, the absorbance of the resulting solution was determined at 700 nm. A higher absorbance at 700 nm suggested better reducing power ability [45].

#### 3.6.2. DPPH Radical Scavenging Activity Assay

DPPH method is a convenient and easy method to determine the antioxidative activity of protein hydrolysates or peptides. The scavenging activity of HAHp-G MRPs on DPPH radicals were evaluated with the method described in our previous study [34]. Briefly, 100 μL of HAHp-G MRPs were diluted into 1.0 mL with distilled water, and mixed with 99.5% ethanol at a ratio of 1:1 (*v/v*). An aliquot 250 μL of 0.02% DPPH (ethanol solution) was added into the mixture. After blending vigorously, the reaction mixtures were kept in the darkness at room temperature for 60 min. The absorbance of the resulting solution (A_S_) was measured at 517 nm using a 722 spectrophotometer (Shanghai Precision Scientific Instrument Co., Ltd, Shanghai, China). The DPPH radical scavenging activity was calculated using the following Equation (2):

radical scavenging activity (%) = [(A_C_ − A_S_ + A_B_/A_C_] × 100 (2)

where A_C_ replaced the sample solution with the same volume of distilled water; A_B_, replaced DPPH solution with the same volume of 99.5% ethanol.

### 3.7. Antibacterial Activity

#### 3.7.1. Determination of Antibacterial Spectra and MIC

The HAHp-G MRPs, which demonstrated strong reducing power and scavenging DPPH radical ability, was selected to assay the antibacterial activity using agar-well diffusion method [35] with few modifications. Briefly, a loop of single bacterial colony of *E. coli*, *P. fluorescens*, *P. vulgaris*, *P. aeruginosa*, *S. aureus*, *B. subtilis*, *B. megaterium,* and *S. lutea* was inoculated into 10 mL of nutrition broth (peptone 10 g/L, beef extract 3.0 g/L, sodium chloride 5.0 g/L, pH 7.2), respectively. After incubation at 37 °C overnight, 100 μL of bacterial suspension (about 10^6^ cells/mL) was spread evenly over the surface of solidified nutrient agar (around 15 mL) in a Petri dish. A sterilized stainless steel punch was used to dig wells (5 mm diameter). An aliquot of 30 μL of HAHp(5.6)-G MRPs was added to each well. The plate was incubated at 37 °C for 24 h, and the inhibitory zone around the well was observed and measured.

The minimal inhibitory concentration (MIC) value was determined using a two-fold microdilution method [63]. Serial dilutions of 100 μL of sample were added into a 96-well plate. Then 100 μL of bacterial suspension (1.0 × 10^6^ cells/mL) was added to each well. After blending, the 96-well plate was incubated at 37 °C for 24 h. The MIC value was defined, at which no visible bacteria were observed with the naked eye or microscope.

#### 3.7.2. Scanning Electron Microscopy (SEM)

The suspensions of *E. coli* (500 μL) at the exponential phase were mixed with sample (500 μL), and incubated at 37 °C for 3 h. After centrifugation for 10 min at 2,000*× g*, the bacterial pellet was washed twice with sterilized 0.8% NaCl. The resulting precipitate was fixed with 3.0% (w/v) glutaraldehyde at room temperature for 12 h. After being rinsed three times with 10 mM sodium phosphate buffer (pH 7.0), the bacterial suspensions were dehydrated in gradient concentrations of ethanol. After critical-point drying and layering with 20-nm gold coating, the microscopy of *E. coli* was observed with an S-3400N scanning electron microscope (Hitachi, Tokyo, Japan).

### 3.8. Determination of Food Functional Properties

#### 3.8.1. Foaming Capacity and Stability

Foaming capacity and stability of the HAHp-G MRPs, which showed high antioxidative activity and antibacterial activity, were determined at various pH values by the method described by Sathe and Salunkhe [64] with few modifications. Briefly, 5mL of sample were adjusted to pH 2, 4, 6, 8, and 10, respectively, and then homogenized at a speed of 16,000 rpm for 2 min. The whipped sample solution was transferred into a 10 mL cylinder. The total volume was read after 30 s. After allowed to stand at 20 °C for 3 min, the volume of whipped sample was measured again. The unheated HAHp-G was used for control group. Foaming capacity (FC) and foaming stability (FS) were calculated using the following Equations (3) and (4):

(3) $$\text{Foaming capacity }\left(\%\right)\;=\frac{Α-Β}{B}\times 100$$

(4) $$\text{Foaming stability }\left(\%\right)\;=\frac{Α-Β}{B}\times 100$$

where A was the volume after whipping (mL); B was the volume before whipping (mL); and C was the volume after standing (mL).

#### 3.8.2. Emulsifying property

Emulsifying capacity and stability of HAHp(5.6)-G MRPs were measured using the method of Song, Wei, and Luo [65]. In brief, the pH of HAHp(5.6)-G MRPs (3 mL) was adjusted to 2, 4, 6, 8, and 10, respectively. An aliquot of 10 mL soybean oil was added to the solution and homogenized at a speed of 20,000 rpm for 1 min. Then an aliquot of 50 μL emulsions was pipetted from the bottom of the container at 0 and 10 min after homogenization, and mixed with 5 mL of 0.1% sodium dodecyl sulfate solution, respectively. The absorbance of the diluted solution was measured at 500 nm. Emulsifying activity index (EAI) and emulsion stability index (ESI) were calculated according to the following Equations (5) and (6), respectively. The EAI and ESI of unheated HAHp(5.6)-G was measured for comparisons.

(5) $$\text{EAI }\left(m^{2}/\text{mL}\right)=\frac{2\times 2.303\times A_{500}}{0.25\times \text{ protein volume}}$$

(6) $$\text{ESI }\left(\text{min}\right)=\frac{A_{0}\times △t}{△A}$$

where ΔA = A_0_ − A_10_, and Δt = 10 min.

### 3.9. Statistical Analysis

All of these tests were performed in triplicate. Data were expressed as means ± standard deviations (*n = 3*). Statistically significant effects were analyzed using the statistical package SPSS 16.0 (SPSS Inc., Chicago, IL, USA). The independent Student’s *t-test* was used to evaluate significant differences (*p* < 0.05) between samples.

## 4. Conclusions

It can be concluded that the Maillard reaction-modified peptic hydrolysates of half-fin anchovy, HAHp-G MRPs, were endowed with better antioxidant activities on reducing power and DPPH radical scavenging than HAHp. In particular, the products of HAHp(5.6)-G MRPs generated at pH 5.6 should be desirable MRPs due to their lower browning indices, which imply fewer possible negative effects derived from MRPs. Furthermore, HAHp(5.6)-G MRPs showed great antibacterial activity and demonstrated excellent foaming capacity and stability, and emulsifying capacity and stability compared to the unheated HAHp(5.6)-G. Therefore, this study shows HAHp(5.6)-G MRPs may be of interest to the seafood industry as a food additive of marine origin.

## Figures and Tables

**Table 1:** Antibacterial activity and MIC of HAHp(5.6)-G MRPs.

Bacterial Strains	Inhibition Zone Diameter (mm)	MIC (μg/mL)
Gram-negative		
*E. coli*	28.2 ± 0.06	8.3
*P. aeruginosa*	13.1 ± 0.00	16.7
*P. vulgaris*	22.1 ± 0.01	8.3
*P. fluorescens*	21.8 ± 0.30	8.3
Gram-positive		
*B. subtilis*	22.8 ± 0.30	8.3
*B. megaterium*	12.5 ± 0.09	16.7
*S. lutea*	11.9 ± 0.01	16.7

## Abbreviations

The following abbreviations are used in this manuscript:

MR: Maillard reaction
MRPs: Maillard reaction products
DPPH: 1,1-diphenyl-2-picrylhydrazayl
HAHp: the peptic hydrolysates of half-fin anchovy
HAHp-G MRPs: the half-fin anchovy hydrolysates-glucose Maillard reaction products
BI: browning intensity
BSA: bovine serum albumin
GSSG: oxidized glutathione
GSH: reduced glutathione
MIC: the minimal inhibitory concentration
SEM: scanning electron microscopy
FC: foaming capacity
FS: foaming stability
EAI: emulsifying activity index
ESI: emulsion stability index
